# Non medical prescribing leads views on their role and the implementation of non medical prescribing from a multi-organisational perspective

**DOI:** 10.1186/1472-6963-11-142

**Published:** 2011-06-02

**Authors:** Molly Courtenay, Nicola Carey, Karen Stenner

**Affiliations:** 1 Faculty of Health and Medical Sciences, University of Surrey, Guildford, Surrey, UK

## Abstract

**Background:**

In the United Kingdom, non-medical prescribing (NMP) has been identified as one way to improve healthcare quality and efficiency. Healthcare organisations are charged with overseeing the clinical governance of NMP and guidance recommends the identification of a lead director to be responsible for its implementation. While over twelve million items are prescribed each year by the 50,000 qualified NMPs its uptake is inconsistent. Several studies have explored the barriers to NMP at a practice level, however little is known about the role the NMP lead and the implementation of NMP from an organisational perspective. The aim of this research was to explore the role of the organisational NMP lead across a range of practice settings within one Strategic Health Authority (SHA) and consider the development of NMP from a multi-organisational perspective.

**Methods:**

Semi-structured telephone interviews with 28 NMP leads across one SHA were undertaken by a trained qualitative researcher. Interviews addressed the purpose of the role and difficulties encountered; audiotapes were transcribed, coded and themes were identified.

**Results:**

The NMP lead role comprised of four main functions; communication, coordinating, clinical governance and support. Factors hampering progress in overseeing the safe development of NMP included lack of clarity about the NMP lead role and responsibilities, strategic support and a lack of protected time. The extent to which clinical governance systems were in place across organisations was inconsistent. Where a strategic approach to its development was adopted, fewer barriers were encountered and NMP was more likely to become embedded within organisations.

**Conclusions:**

The significant contribution that NMP leads play in embedding NMP within organisations should be acknowledged by clearer national guidance for this role and its responsibilities. Greater standardisation and consistency is required of clinical governance systems if quality and safety is to be ensured given the expanding development of NMP. The extent to which NMP is in place worldwide differs. However, our findings will be of interest to policymakers in other countries involved in the development and implementation of this role.

## Background

As part of the modernisation of the United Kingdom (UK) healthcare workforce, prescribing authority has been extended to nurses, pharmacists, allied health professionals (AHPs) (including radiographers, physiotherapists, podiatrists/chiropodists) and optometrists [1,2]. Although prescribing by non medical healthcare professionals is in place in some countries (such as the States and Sweden) and anticipated in others (e.g. the Netherlands) [3], non medical prescribers (NMPs) in the UK have the most extended prescribing rights in the world. They offer a strategic innovative solution to address capacity, quality, efficiency, and effectiveness if used widely in pathway redesign and should be considered within all service specifications where medicines are prescribed or supplied [2]. There are now over 50,000 NMPs across the UK. This includes more than 30,000 community practitioner prescribers with powers to prescribe from a restricted formulary (mainly over-the-counter medicines and wound dressings) and approximately 19,000 nurses [4], 2,000 pharmacists, and several hundred AHPs and around 70 optometrists [5] with an independent [2] and/or supplementary prescribing [6] qualification. Over 12 million items are prescribed each year by NMPs in primary care [7] and the extension of prescribing rights to paramedics and other groups of AHPs (including dieticians) is currently under consideration [8,9].

Independent Prescribing (IP) and Supplementary Prescribing (SP) are two different forms of prescribing. Through IP, nurses and pharmacists may assess, diagnose and prescribe independently any licensed or unlicensed medicines with the exception of controlled drugs (CDs). Under current legislation [3], nurses can prescribe some CDs whereas pharmacists cannot. Supplementary prescribing, in contrast, is a form of dependent prescribing where the initial assessment and diagnosis is carried out by a doctor and the medicines to be prescribed are detailed in a Clinical Management Plan (CMP) agreed between the doctor, NMP and patient. Through SP, the NMP can prescribe any medicine including CDs.

Learning on the NMP programme is often between nurses, pharmacists and AHPs (optometrists undergo separate training). The programme comprises 27 taught days (although some programmes have a distance learning element) and 12 days in practice with a designated medical practitioner (DMP) who is responsible for the assessment of practice [2]. On completion of the course, nurses and pharmacists are awarded a dual qualification in independent and supplementary prescribing whereas AHPs qualify as supplementary prescribers.

There is evidence available that NMPs can improve the quality of care patients receive and provide services that are both flexible and accessible to patients. For example, patients value the interpersonal skills of nurse and pharmacist prescribers and the longer consultations they offer [10-14]. Improved access to medicines and increased service efficiency are benefits attributed to NMP by patients, NMPs, doctors and other stakeholders [10,15,16]. Nurses and pharmacists have been shown to be competent at assessing patients, producing appropriate prescriptions, and providing patients with information and advice about treatment and side effects [10,17,18]. Adopting the prescribing role is reported to increase job satisfaction, improve inter-professional collaboration and encourage flexible team working [10,19]. In some areas, such as nurse-led dermatology clinics, NMP has enabled the development of new services with less dependence on doctors [16]). However, despite these reported benefits, the uptake and use of NMP is inconsistent. For example, while high numbers of nurses in general practice prescribe [20], poor uptake/and or use has been reported by pharmacists [21], nurses working in diabetes services [22], and mental health [23].

Unsuccessful implementation of NMP is potentially costly in terms of the time and expenses for training individuals to prescribe [10] and failure to deliver predicted efficiency savings within services. A number of studies have explored the barriers to NMP at a practice level. Barriers with regards to planning for NMP include the cost of training [10] and a lack of incentive to undertake it [15]. The initiation of prescribing once qualified can be prevented by a lack of confidence on behalf of the prescriber, a lack of clarity over the prescribing role, difficulties in accessing medical records and electronic prescriptions, inadequate procedures for registering new prescribers and obtaining prescribing pads [20,24-26]. Restrictions imposed by national and local regulations and restrictions set by individual trusts, resistance from doctors or managers, lack of access to a prescribing budget, inadequate support, access to continuing professional development (CPD) [20,26], and practical difficulties in implementing the CMPs necessary for supplementary prescribing [27,28], can each influence prescribing activity and its on-going use.

Guidance [2] refers to the responsibilities of organisations to develop a strategic plan for NMP and this plan should include the identification of a lead director responsible for the implementation of NMP within the organisation. To ensure that NMP is used safely and efficiently it should be included within organisational clinical governance arrangements [2]. However, there is no evidence available that has explored the development of NMP from an organisational perspective. The aim of this research was to explore the role of the organisational NMP lead across a range of practice settings within one Strategic Health Authority (SHA) and consider the development of NMP from a multi-organisational perspective.

## Methods

### Design

A qualitative design was selected, using semi-structured telephone interviews and framework analysis.

### Participants

All NMP leads across the SHA (n = 44) were contacted via email (with a reminder two weeks later), and invited to participate.

### Data collection

A two-part interview schedule was developed based on previous work in the area [29,30] and comments from the project steering group. The first part asked participants for details about their role (i.e. job title, whether their role was a clinical role and if they were a prescriber), if they had designated time for the lead role and length of time in post, the number and types of prescribers they covered (including area of practice/geographical area) and, if they held a database of prescribers details, what information they stored. Participants were then asked the extent to which safety and clinical governance procedures were in place. The second part of the interview asked participants about the purpose of their role, difficulties encountered, and factors supporting their role.

Interviews, held at mutually convenient times and lasting between 25-45 minutes, were conducted between August 2009 and December 2009.

### Ethical consideration

NHS and University Ethics Committee approval for the study was obtained. NMP leads who were interested in participating in the study were provided with an information sheet and given the opportunity to ask the researchers any questions. Participants were informed that the interview would be conducted in two parts with part 1 recorded on paper, and part 2 audio-taped, transcribed and anonymised. Consent was obtained prior to interviews and participants were free to withdraw from the study at any time.

### Data Analysis

SPSS version 17 was used for data entry and analysis of part 1 of the interview. Data was summarized using descriptive statistics. Qualitative data generated in part two of the interview were analysed using a form of framework analysis [31]. Interview transcripts were read by two researchers who then worked together to develop a coding framework based on a combination of emerging themes and initial research questions. The researchers then applied the framework to code each interview transcript using ATLAS ti software and then charted the range and variety of responses under each code. Once charted, patterns and associations in the data were identified and organised into themes. The two researchers initially worked independently to analyse different interviews, meeting regularly to discuss changes to the coding framework before working together to develop the final analysis.

## Results

Twenty eight (64%) of the 44 NMP leads across the SHA agreed to participate. They worked in hospital, primary care and mental health trusts and were responsible for varying numbers and types of prescribers. Characteristics of the leads and their role are summarised in table 1. Participants held a mixture of managerial and clinical roles, eight of which were at directorate level. Fourteen (50%) participants held a prescribing qualification. Nineteen (68%) reported that there was no designated time for the role and 15 (54%) spent between 30-60 minutes a week on it.

**Table 1:** Non Medical Prescribing Leads and their Role

	Yes		No		Not applicable	
***n = 28 unless otherwise stated***	*n*	%	*n*	%	n	%

**Job Title**						

Clinical quality & governance	4	14.3				

Strategic pharmacist	3	10.7				

Chief/deputy chief nurse	8	28.6				

Nurse consultant/clinical nurse specialist	9	32.1				

Education/workforce development	4	14.3				

**Is this a clinical role?**	14	50	14	50		

**Is there designated time for NMP lead role?**	9	32.1	19	67.9		

**Number of hours per week spent on NMP lead role?**					Mean 2.2	

0.5-1	15	53.6			Median 1.0	

1.5-3	7	25.0			Mode 1.0	

>3	6	21.4			Min 0.5-Max 10	

**Do you have a prescribing qualification?**	14	50.0	13	46.4	1	3.6

Nurse independent supplementary prescriber	12	42.9				

Community practitioner prescriber	2	7.1				

**Do you currently prescribe? (n = 14)**	8	57.1	6	43.8		

**How long NMP lead?**						

<6/12-1 year	6	21.4				

2-5 years	14	50.0				

>5 years	8	28.6				

**Area of practice?**						

PCT	10	35.7				

NHS trust	11	39.3				

Mental Health	6	21.4				

PCT/NHS Trust & Mental Health	1	3.6				

**Geographical area?**						

Rural	4	14.3				

Urban	2	7.1				

Mixed	21	75.0				

Not applicable	1	3.6				

**Do you hold a current database?**	25	89.3	1	3.6	2	7.1

**Information on database? (n = 26)**						

Types of prescriber	26	100				

Registration details	24	85.7	2	7.1		

Work setting or clinical area	23	82.1	3	10.7		

If they are prescribing	18	64.3	8	28.6		

If they are not prescribing	15	57.6	11	42.4		

Other (CRB check, supervisor details, manager details, audit)	8					

**Number of prescribers lead is responsible for**	Range: 3 -547		Mean: 87.3			

**Types of prescribers covered by NMP lead**						

Community practitioner prescriber	18	64.3				

Pharmacist independent/supplementary prescriber	21	75.0				

Nurse independent/supplementary prescriber	22	78.6				

AHP (podiatrist/physiotherapist/radiologist)	6	21.4				

Optometrist	2	7.1				

(NMP = non-medical prescribing, PCT = Primary Care Trust, NHS = National Health Service, CRB = Criminal Records Bureau, AHP = allied health professional)

### The role of the lead in supporting NMP

Participants reported that their role had four core elements (see table 2) each of which is explored below:

**Table 2:** Key aspects of the NMP lead role

**Information and communication **(n = 21)
➤ Two-way communication between trusts and NMPs (n = 21)
➤ Information conduit (n = 18)
○ a) keep up to date with national policy, legal issues & good practice
○ b) disseminate information on national policy, legal issues & good practice to NMPs
➤ Point of contact (n = 11)

**Promoting and co-ordinating **(n = 17)
➤ Ensuring that applicants meet NMP selection criteria (n = 11)
➤ Co-ordinate and promote NMP within trust (n = 17)
➤ Work to integrate and expand NMP in service planning (n = 10)
➤ Liaise with education providers to ensure NMP programme meets needs of employees (n = 5)
➤ At a strategic level raise awareness and profile of NMP in less developed areas of practice (n = 3)

**Clinical governance **(n = 22)
➤ Ensure clinical governance systems are in place and up to date (n = 22)
➤ Monitor NMP practice (n = 21)
➤ Identify and deal with NMP related governance issues (n = 22)
➤ Monitor and support those not using prescribing qualification (n = 4)

**Support and training **(n = 17)
➤ Support NMPs before, during and after implementation of NMP in practice (n = 17)
➤ Support supervisors of NMPs (n = 17)
➤ Provide information about continuing professional development opportunities (n = 10)
➤ Provide practical training session and support groups for NMPs (n = 10)
➤ Active support role during initial implementation of NMP into practice (n = 8)
➤ Provide broad medicines management support to all NMPs (n = 1)

(NMP = non-medical prescribing)

#### i) Information and communication

One of the main elements in which leads were involved was two-way communication between the trust and NMPs. Participants described how they acted as an information conduit, disseminating information to NMPs on legislation, policy and good practice so as to ensure that they were kept up-to-date. They also described how they were a point of contact for NMPs to discuss any prescribing issues or queries and how they fed this information back to the trust

*'I think it's (my role) to be a conduit so that information coming into the organisation gets to all the NMPs because they're all so diverse and in so many different little pockets and silos. I see my prime responsibility is to make sure that they're kept up to date with any change. If I get access to information about prescribing practice nationally or, the East of England, I see that it gets to them.'*[19]

#### ii) Promoting and co-ordinating

Promoting and co-ordinating NMP within the trust was also considered to be a key aspect of the lead role as was working to integrate and expand NMP into service planning.

*'It's actually making sure that we are driving this agenda, giving nurses opportunities, looking at new ways of working, trying to improve patient care and giving you know patients, you know benefits to prescribing.'*[5]

A number of leads felt that in order to raise the profile of NMP in less developed areas, for example pharmacist prescribing, that their involvement in decision making at a strategic level was necessary. Liaising with higher education providers to ensure the NMP programme met the learning needs of their employees was also considered to be an important aspect of the leads role by several participants.

*'to inform the pharmacy networks on regional NMP initiatives, to provide input from a pharmacy perspective about where pharmacist prescribing can fit into care pathways as an alternative provider and to ensure that the pharmacy workforce (being very small) is not forgotten as potential prescribers for particular care pathways in different sectors.'*[11]

#### iii) Clinical governance

Ensuring that clinical governance systems were in place and up-to-date was felt to be a critical part of the role by the majority of leads as described in the quote below:

*"..from an organisational perspective that we are assured that the organisation provided a safe environment for the prescribers to prescribe in and that ...we have systems and processes in place that would fulfil the accountability role, the support role, the development role." *[13]

All but one lead held an electronic database in which they recorded the details of NMPs i.e. type of prescriber, work setting, and whether or not the prescriber prescribed. However, the extent to which this information was recorded by NMP leads varied (see table 1). Table 3 provides a detailed summary of the extent to which leads reported each aspect of clinical governance was in place. Systems were reported to be in place for ordering and distribution of the British National Formulary (BNF) (and the Nurse Prescribers' Formulary (NPF) for Community Practitioners and the Drug Tariff), ensuring NMP policies were updated and that NMPs kept informed of relevant clinical information. However, the extent to which systems for monitoring prescribing were in-situ, and the degree to which practitioners were able to access monitoring data and participate in clinical audit and review was inconsistent. Difficulties were reported to be encountered in monitoring private practitioners (such as community pharmacists and NMPs working in the private sector), deciding how best to govern non-active NMPs or those who seldom prescribed, and determining responsibility for governing NMPs employed in general practice.

**Table 3:** The extent to which NMP leads report safety and clinical governance systems are in place

*Totals exclude respondents who indicted this was not applicable to their role*	Yes		No	
	*n*	%	*n*	%

1. Is there a system in place for ordering and distribution of the BNF, the NPF for Community Practitioners and the Drug Tariff? (n = 27)	27	100	0	0

2. Do you monitor NMP legislation and ensure that policies are updated in line with the revised legislation? (n = 26)	25	96.2	1	3.8

3. Do mechanisms exist to ensure all NMPs are kept informed of relevant clinical information, e.g. Patient Safety Notices, Drug Alerts and Hazard Warnings? (n = 27)	25	92.6	2	7.4

4. Is there an up-to-date NMP policy in place? (n = 27)	24	88.8	3	11.2

5. Is there is a mechanism in place to ensure the selection of suitable candidates for NMP training? (n = 27)	24	88.8	3	11.2

6. Is there a system in place for the acquisition and retention of specimen signatures to identify prescribers? (n = 27)	23	85.2	4	14.8

7. Are NMPs receiving appropriate support or supervision in their prescribing role (e.g. local clinical supervision groups/learning sets or peer-support groups)? (n = 27)	22	81.5	5	18.5

8. Do NMPs have an agreed scope of practice or equivalent and is a copy of this retained by the organisation? (n = 26)	21	80.8	5	19.2

9. Do NMPs identify and fulfil continuing professional development needs relevant to their clinical work? (n = 25)	20	80.0	5	20.0

10. Are systems for monitoring prescribing (e.g. PACT data) in place in all sectors of practice? (n = 27)	18	66.7	9	33.3

11. Are NMPs involved in the development of local formularies and guidelines e.g. drug and therapeutic committee? (n = 26)	17	65.4	9	34.6

12. Do NMPs participate in regular clinical audit and reviews of their clinical services? (n = 23)	15	65.2	8	34.8

13. Do practitioners in all areas of practice have access to monitoring data? (n = 27)	14	51.9	13	41.8

(NMP = non medical prescribing, BNF = British National Formulary, NPF = Nurse Prescribers Formulary, PACT = Prescription analysis and cost trend)

#### iv) Support and training

A number of leads felt that their role had a supportive element both towards NMPs and their DMPs. Several leads were involved directly and indirectly in providing CPD to NMPs in their trust. Whilst some leads were mainly involved with providing information about available training, others had a more active role whereby they were directly responsible for running training sessions in their trust.

*'I put on half day support session updating their physical examination skills and to network amongst themselves and we bring in...I ask them to bring a clinical issue, something that's happened to them that they'd like to share.'*[6]

The extent to which they were involved in these elements varied, for example under half (n = 10) of the participants provided support and training directly to NMPs.

### Strategic vision

Barriers and facilitators to the development and support of NMP reported by leads are detailed in table 4.

**Table 4:** Barriers and Facilitators to non-medical prescribing

	Facilitators	Barriers
**Justifying need (n = 14)**	❖ Trust strategy & commitment to promote and fund NMP ❖ Expectations of course and intended NMP role are discussed with interested candidates and their DMPs. Having a defined set of patients/conditions e.g. role as specialist nurse	❖ Lack of strategic approach in organisations ❖ Lack of support from managers & clinicians ❖ Overly restrictive trust strategy to expanding the number of NMPs ❖ Lack of vision and/or evidence of benefits of commissioning services in new and developing areas e.g. community based pharmacists

**Finding a practice supervisor (n = 18)**	❖ Having an established relationship with potential DMP ❖ NMP lead support for inexperienced DMPs ❖ DMPs who have already been a mentor and have positive experience of NMP	❖ Lack of support when developing NMP in new areas of practice ❖ Lack of financial incentive to act as DMP ❖ NMP candidates who have to find and secure DMP support in different setting to their usual area of practice, e.g. those working across a number of GP practices

**Preparation for prescribing role (n = 23)**	❖ Systematic and structured approach to selecting students for NMP training, e.g. use of national criteria ❖ Trusts who provide additional training to ensure students have pre-requisites for NMP training e.g. numeracy training, assessment and diagnostic training, mental health pharmacology module ❖ Students are prepared for the prescribing programme with respect to course content & amount of learning that is required ❖ Having a well defined prescribing role that is agreed between NMP and their manager	❖ Inconsistent approach to selection process ❖ Lack of awareness (amongst candidates and managers) of NMP course academic content and requirement ❖ Inappropriate expectations (amongst candidates, manager or clinicians) with respect to remuneration and how prescribing qualification will be used in practice ❖ Relevance of NMP programme to non-community based nurses ❖ Inconsistent methods of academic assessment of NMP between different education providers

**Confidence & ongoing support (n = 19)**	❖ Trust provision of NMP support groups, meetings and networks ❖ NMPs receive support (from NMP lead, DMP or Peers) during initial implementation and role transition ❖ NMPs receive ongoing support from other NMPs and their own clinical team (including clinical supervision) ❖ Supplementary prescribing used as means to build confidence	❖ A lack of support approach within trust ❖ Lack of understanding about, and access to appropriate CPD for prescribing role ❖ Providing support for community & mental health based NMPs ❖ A lack of confidence to negotiate prescribing responsibility within mental health settings or problems defining individual scope of practice ❖ Restrictions imposed by enforced use of supplementary prescribing

**Practicalities & legalities (n = 11)**	❖ Procedures for registering and governing NMP up-and-running in organisation	❖ Confidence reduced by the time lag between course completion, registration with professional body as NMP, and implementation of role ❖ Implementing NMP across range of providers in primary & secondary care

(NMP = non medical prescribing, DMP = designated medical practitioner, GP: general practice, CPD = continuing professional development)

The amount of difficulty encountered depended upon the strategic approach of the trust towards NMP and the involvement of the NMP lead. The extent to which organisations had a clear strategy varied. Where a consistent strategic approach to developing NMP was in place, greater consideration was given to processes including workforce planning, the selection of candidates for prescribing training, the provision of clinical supervision, ongoing support, continuing professional development, and organisational preparation for the role in terms of procedures for registering as a prescriber and initiating prescribing. For example, it was a requirement by some specialist services, that those delivering the service had the prescribing qualification. New staff appointed to that service were therefore automatically put forward for prescribing training:

*'.....thinking more around some specialist services we have like x condition it's written within the service specification that whoever is delivering that service requires the prescribing qualification and so I've just had a new person come into that team and they've now put forward to go on the prescribing course.'*[10]

The extent that individuals were involved in the various lead role activities varied, but greater involvement was instrumental in alleviating many of the barriers. For example, involvement in the selection process ranged from no input to stringent interviewing of all candidates. Procedures for developing NMP varied: some candidates self-selected, some were selected by their manager, and some via strategic workforce planning (i.e. where having an NMP qualification was written into job descriptions for certain roles). Selection criteria also varied (e.g. some trusts set a minimum job banding and three participants were uncertain if criteria were in place).

*'it varies across the Trust because it varies according to the area []initially it was a case of nurses who were interested in going forward for the qualification would apply and then if it was appropriate they'd be assessed, is it appropriate for their role, were they going to be able to work to the academic level required and then it would be... would the service actually able to free them up for the training [] but what we've done in community nursing is that because we've reviewed the education pathway we're now making independent prescribing an essential component of the specialist training so in the future all nurses going forward who come out at the other end as a Community Nursing Sister [], will have the independent prescribing qualification.' *[20]

However, leads who discussed prescribing role expectations with potential NMPs, and followed criteria to select individuals with the appropriate skills and experience, said this helped identify and address problems at an early stage and so reduce barriers to prescribing once qualified. Providing support or requiring completion of additional courses (e.g. on numeracy, assessment and diagnostics, pharmacology and mental health assessment) pre-empted problems during and post training. Problems securing ongoing support, finding a DMP, and defining NMP roles were alleviated where leads provided support and discussed expectations with new DMPs, NMP candidates and managers.

Lack of strategic vision to develop NMP reflected a lack of organisational support, making it hard for individuals to justify the need to train. Equally, an overly restrictive selection procedure presented barriers to expanding NMP, especially within new areas of practice. Difficulties were reported in gaining approval to develop NMP for candidates without a defined specialty. Five (19.2%) participants reported they were unaware whether NMPs had an agreed scope of practice. Defining scope of practice was particularly difficult for community pharmacists:

*'If there isn't a commissioned service available, the primary care trust (PCT) will not provide the prescriber with an NHS prescribing patch. If the training was part of that process (commissioning), they would obviously have less of an issue about that. But if they were doing it off their own back, they would have to write a business case and persuade the PCT to support that pathway.' *[11]

Agreement was easier for individuals who worked within well-defined areas within general practice or in specialist practice areas. In these instances, NMPs were said to be well supported and actively prescribed.

### Factors supporting the NMP lead role

Despite the lack of designated time for the role, leads generally reported to be well supported within their organisations, with the exception of two leads who felt there was a lack of strategic support for NMP within the trust. Although a lack of guidance and support at a local and strategic level, misunderstanding of the NMP role by clinicians, managers or trusts acted as barriers to the NMP lead role (see table 5), the role was supported by several factors including knowledge and experience of NMP, and having good relationships with directors, members of executive teams, colleagues and pharmacists:

**Table 5:** Factors supporting the NMP lead role

Supportive Factors	Areas of difficulty
• Good relationships with colleagues, pharmacists, members of executive teams and directors. • Knowledge of NMP • Experience as a NMP • Knowledge and experience of trust wide issues e.g. clinical governance, management and legal issues • Guidance from SHA • Good relationships with external educational organizations • Established policies and procedures for: a) identifying the need for NMP development within the trust, b) rigorous selection procedure c) clinical governance procedures for NMP	• A lack of leadership at both a national and strategic health authority level • Misunderstanding of the NMP role by clinicians, managers or trusts • Lack of support for medicines management, auditing and monitoring information on prescribing data • Lack of guidance over action to be taken over NMPs who are not prescribing, or prescribe infrequently • Poor communication, lack of support and guidance from individual trusts • Lack of clarity about the duties of the NMP lead role • A lack of designated time for lead role, large variations in geographical coverage, range of NMP roles and problems integrating a range of systems • Having little choice or control over the quality of education provision • Achieving and maintaining attendance at trust wide NMP meetings. • Finding professional support for the NMP lead role

(NMP = non medical prescribing, SHA = Strategic Health Authority)

*'Yes we have a Pharmacy Advisor who attends our meetings, attend the non-medical prescribing meeting, you know supports us and also give us the Prescription Analysis and Cost Trend (PACT) data information and analyse as to what people's prescribing patterns are like so that we can, you know, analyse it and then discuss it, you know groups and individuals as to you know, what the issues are with prescribing and also the prescribers can phone the Pharmacy Advisors in the PCT and ask for support and information in practice when they are prescribing'. *[14]

## Discussion

This study is the first to specifically explore the role of the NMP lead from a range of practice settings and consider the development of NMP from a multi-organisational perspective. We acknowledge that the study is limited to participants from one SHA.

The findings demonstrate the important role that the NMP lead plays with regards to supporting and overseeing the development of NMP within NHS trusts. However, a lack of consistency and clarity about the function of the NMP lead role, coupled with poor strategic support for NMP within some organisations, hampered progress. While some worked at directorate level, others had a clinical or educational remit. A lack of dedicated time for undertaking this role, inconsistencies in responsibilities, and large variations in coverage were reported. The number of NMPs and size of area covered impacted on the amount of time required to carry out the role, however these factors did not appear to have been considered by trusts when allocating lead roles. It is evident from our findings that the NMP lead role comprises several core functions including information and communication, promoting and coordinating NMP, clinical governance and support and training. Guidance is required from managers with regards to these functions and sufficient time dedicated to the role in order that they can be successfully achieved. Those factors which support leads in their role including good relationships with colleagues, knowledge and experience of NMP and trust wide issues, guidance from the SHA, and established policies and procedures should also be borne in mind.

Patient safety and quality care is a priority for all health care professionals. NMPs provide a broad range of services enabling patients' easier access to medicines and increase choice in accessing medicines. It is essential that this is conducted safely and effectively within robust clinical governance frameworks [2]. Clinical governance procedures were in place for recording prescribers details, disseminating important communications, updating policy and ordering and distributing the BNF and related documents to prescribers. However, systems for monitoring, clinical audit and review were not reported to be consistently in place across all sectors of practice. This pattern of NMP governance is similar to that reported elsewhere [29,30]. These findings emphasise that development is required to support systems for monitoring and audit and to ensure that all NMPs can review their own prescribing data, including where electronic data is not readily available. Further difficulties were encountered in determining governance arrangements and monitoring for private practitioners and NMPs in general practice. These difficulties are likely to increase under recently proposed policy changes [32].

Factors reported as acting as barriers to NMP including justifying a need to prescribe, finding a practice supervisor, preparation for the role, on-going support once qualified, and practicalities and legalities are in-line with those identified previously [33-35]. Consistent with the findings of two recent studies evaluating pharmacist prescribing [36] and prescribing in mental health [23], a lack of clear strategy at an organisational level was a major barrier to NMP in this study. Although difficulties were experienced gaining support for NMP for those working in new areas of practice and without a defined specialty, where strategies were in place, many of these difficulties were overcome as NMP was more likely to become embedded within organisations (through inclusion in workforce planning, selection processes, training, support and organisational preparedness); the NMP lead playing a significant role in each of these processes.

## Conclusions

The significant contribution that NMP leads play in embedding NMP within organisations should be acknowledged by clearer national guidance for the role, its responsibilities and workload. While procedures and policy for monitoring NMP are in place within NHS trusts, greater standardisation and consistency is required of clinical governance systems if quality and safety is to be ensured given the expanding development of NMP. The extent to which NMP is in place worldwide differs. However, our findings will be of interest to policymakers in other countries involved in the development and implementation of this role.

## Competing interests

The authors declare that they have no competing interests.

## Authors' contributions

MC was responsible for the study conception and design. NC performed the data collection and analysis. KS helped with the data analysis. All authors participated in the drafting of this manuscript and have approved the final manuscript.

## Pre-publication history

The pre-publication history for this paper can be accessed here:

http://www.biomedcentral.com/1472-6963/11/142/prepub

## References

[B1] DoH The NHS Plan A Plan for Investment, a Plan for Reform 2000 London: DoH

[B2] DoH Improving patient's access to medicines: A guide to implementing nurse and pharmacist independent prescribing within the NHS in England 2006 London: DoH21638234

[B3] Ball J Implementing nurse prescribing 2009 Switzerland: International Council of Nurses

[B4] Culley F Professional considerations for nurse prescribers Nursing Standard 2010 24 43 55 60 20669699 10.7748/ns2010.06.24.43.55.c7880

[B5] DoH Verbal Communication 2010

[B6] DoH Supplementary Prescribing 2005 London: DoH

[B7] NHS Prescription Service Report Update on growth in prescribing volume and cost in the year 2010 London: NHS Prescription Service

[B8] DoH Proposals to introduce prescribing responsibilities for paramedics: stakeholder engagement 2010 Consultation paper. London: DoH

[B9] DoH Allied health professions prescribing and medicines supply mechanisms scoping project report 2009 London: DoH

[B10] issell P Cooper R Guillaume L Anderson C Avery A Hutchinson A James V Lymn J Marsden E Murphy E Ratcliffe J Ward P Woolsey I An evaluation of supplementary prescribing in nursing and pharmacy Final report 2008 DoH

[B11] Brooks N Otway C Rashid C Kilty E Maggs C The patient's view: the benefits and limitations of nurse prescribing British Journal of Community Nursing 2001 6 7 342 348 11865224 10.12968/bjcn.2001.6.7.7066

[B12] Page D Grant G Maybury C Introducing nurse prescribing in a memory clinic Dementia 2008 7 1 139 160 (2008) 10.1177/1471301207084393

[B13] Courtenay M Stenner K Carey N The views of patients with diabetes about nurse prescribing Diabetic Medicine 2010 27 1049 1054 10.1111/j.1464-5491.2010.03051.x 20722679

[B14] Courtenay M Carey N Stenner K Lawton S Peters J Patients views on nurse prescribing: Effects on care, concordance and medicine taking British Journal of Dermatology 2010 164 2 396 401 10.1111/j.1365-2133.2010.10119.x 21054336

[B15] Ryan-Woolley BM McHugh GA Luker KA Prescribing by specialist nurses in cancer and palliative care: results of a national survey Palliative Medicine 2007 21 273 277 10.1177/0269216307079047 17656402

[B16] Carey N Stenner K Courtenay M Stakeholder views on the Impact of Nurse Prescribing on Dermatology Services Journal of Clinical Nursing 2010 19 3-4 498 506 10.1111/j.1365-2702.2009.02874.x 19747199

[B17] Courtenay M Stenner K Carey N An exploration of the practices of nurse prescribers who care for people with diabetes: a case study Journal of Nursing and Healthcare of Chronic Illness 2009 1 311 320 10.1111/j.1752-9824.2009.01034.x

[B18] Courtenay M Carey N Stenner K Nurse prescriber-patient consultations: a case study in dermatology Journal of Advanced Nursing 2009 65 6 1207 1217 10.1111/j.1365-2648.2009.04974.x 19374682

[B19] Stenner K Courtenay M Benefits of nurse prescribing for patients in pain: nurse's views Journal of Advanced Nursing 2008 63 1 27 35 10.1111/j.1365-2648.2008.04644.x 18503536

[B20] Courtenay M Carey N Nurse Independent Prescribing and Nurse Supplementary Prescribing: Findings from a national questionnaire survey Journal of Advanced Nursing 2008 61 4 403 241 10.1111/j.1365-2648.2007.04534.x 18197863

[B21] George J McCaig D Bond C Benefits and challenges of prescribing training and implementation: perceptions and experiences of Royal Pharmaceutical of Great Britain Prescribers Int J Pharm Pract 2007 15 23 30 10.1211/ijpp.15.1.0005

[B22] James J Gosden C Wincourt C Diabetes specialist nurses and role evolvement: a survey by Diabetes UK and ABCD of specialist diabetes services Diabetes Medicine 2009 26 10.1111/j.1464-5491.2009.02716.x 19646199

[B23] Dobel-Ober D Brimblecombe N Bradley E Nurse prescribing in mental health: national survey Journal of Psychiatric and Mental Health Nursing 2010 17 487 493 10.1111/j.1365-2850.2009.01541.x 20633075

[B24] Jones K Edward M While A Nurse prescribing roles in acute care: An evaluative case study Journal of Advanced Nursing 2011 67 1 117 126 10.1111/j.1365-2648.2010.05490.x 21073502

[B25] Otway C The development needs of nurse prescribers Nursing Standard 2002 16 18 33 3812271614

[B26] Carey N Courtenay M Nurse Supplementary Prescribing for patients with diabetes: a national questionnaire survey Journal of Clinical Nursing 2008 17 2185 2193 10.1111/j.1365-2702.2007.02238.x 18705738

[B27] Stenner K Courtenay M A qualitative study on the impact of legislation on the prescribing of controlled drugs by nurses Nurse Prescribing 2007 5 6 257 261

[B28] Stenner K Carey N Courtenay M Implementing nurse prescribing: a case study in diabetes Journal of Advanced Nursing 2010 66 3 522 531 10.1111/j.1365-2648.2009.05212.x 20423387

[B29] Nicholls J Survey of organisational arrangements for non-medical prescribing - summary of results 2009 London Strategic Health Authority

[B30] Healthcare Commission The Best Medicine: the management of medicines in acute and specialist trusts London: Commission for Healthcare Audit and Inspection 2007

[B31] Ritchie J Spencer L Bryman A. and Burgess, R Qualitative data analysis for applied policy research Analysing qualitative data 1993 London: Routledge 173 194

[B32] DoH Equity and Excellence: Liberating the NHS 2010 DoH

[B33] Carey NJ Stenner KL Courtenay M Stakeholder views on the implementation of nurse prescribing in dermatology services Journal of Clinical Nursing 2009 19 498 506 19747199 10.1111/j.1365-2702.2009.02874.x

[B34] Cooper R Anderson C Avery T Stakeholders views of UK nurse and pharmacist supplementary prescribing J Health Serv Res Policy 2008 13 4 215 221 10.1258/jhsrp.2008.008004 18806179

[B35] Hall J Cantrill J Noyce P Why don't trained community nurse prescribers prescribe Journal of Clinical Nursing 2006 5 403 412 10.1111/j.1365-2702.2006.01227.x 16553753

[B36] Baqir WA Clemerson J Smith J Evaluating pharmacist prescribing across the north east of England BJ Clin Pharm 2010 2 147

